# Hydrogen and Deuterium Solubility, Diffusivity and Permeability from Sorption Measurements in the Ni_33_Ti_39_Nb_28_ Alloy

**DOI:** 10.3390/molecules28031082

**Published:** 2023-01-21

**Authors:** Oriele Palumbo, Francesco Trequattrini, Silvano Tosti, Alessia Santucci, Annalisa Paolone

**Affiliations:** 1 Istituto dei Sistemi Complessi, Consiglio Nazionale delle Ricerche, Piazzale A. Moro 5, 00185 Rome, Italy; 2 Dipartimento di Fisica, Sapienza Università di Roma, Piazzale A. Moro 5, 00185 Rome, Italy; 3 Dipartimento Fusione e Tecnologie per la Sicurezza Nucleare, ENEA, Via E. Fermi 45, 00044 Frascati, Italy

**Keywords:** Ni-Nb-Ti alloys, hydrogen solubility, hydrogen diffusion, hydrogen permeability, hydrogen isotope effect

## Abstract

The hydrogen/deuterium sorption properties of Ni_33_Ti_39_Nb_28_ synthesized by the vacuum induction melting technique were measured between 400 and 495 °C for pressure lower than 3 bar. The Sieverts law is valid up to H(D)/M < 0.2 in its ideal form; the absolute values of the hydrogenation/deuteration enthalpy are ΔH(H_2_) = 85 ± 5 kJ/mol and ΔH(D_2_) = 84 ± 4 kJ/mol. From the kinetics of absorption, the diffusion coefficient was derived, and an Arrhenius dependence from the temperature was obtained, with E_a,d_ = 12 ± 1 kJ/mol for both hydrogen isotopes. The values of the alloy permeability, obtained by combining the solubility and the diffusion coefficient, were of the order of 10^−9^ mol m^−1^ s^−1^ Pa^−0.5^, a value which is one order of magnitude lower than that of Ni_41_Ti_42_Nb_17_, until now the best Ni-Ti-Nb alloy for hydrogen purification. In view of the simplicity of the technique here proposed to calculate the permeability, this method could be used for the preliminary screening of new alloys.

## 1. Introduction

The purification of hydrogen to an ultrapure grade (at least 99.9999% hydrogen purity) can be obtained by means of well-conceived metallic membranes [1,2,3]. The principle of operation of these devices is conceptually quite simple: a flux of gases containing hydrogen impacts on the first surface of the metallic membrane, which is able to dissociate the H_2_ molecule; the obtained hydrogen atoms are absorbed into the material and diffuse through it thanks to the difference in the partial pressure on the two surfaces of the membrane; they recombine on the second surface of the material and can be released outside [4]. This process is selective for hydrogen so that high purity of the flux of hydrogen emitted from the second surface is obtained. In order to have an effective and fast process, the membranes should be made of a metal that is able to both absorb and diffuse hydrogen.

The standard material for the production of hydrogen purification membranes is Pd_77_Ag_23_ [1,2,3,5,6], which conjugates the high hydrogen permeability of Pd with the resistance to embrittlement given by the alloying with silver. Although Pd–Ag reactors are an industrial reality, in the past years, there have been many attempts to find different materials with similar permeation properties but comprising less noble, less critical and less expensive metals [1,2,3]. Many materials have been proposed, mainly derived from V [7], Ta [8] and Nb [9], which, as single elements, exhibit permeation properties higher than those of pure Pd [10]. Due to the high solubility of hydrogen in the pure metals and the consequent embrittlement, all metals were used in alloyed forms to improve their mechanical stability.

Among various alloys, Hashi et al. [11] proposed a Ni-Nb-Ti alloy that exhibited a peculiar microstructure composed of two different phases: a bcc NbTi alloy and the eutectic phases NiTi and NbTi [11]. The NbTi phase makes a large contribution to the high hydrogen permeation of the material, while the eutectic phases are able to mitigate the hydrogen brittleness of the Ni-Nb-Ti alloy [11]. Thanks to these specific properties, Ni_30_Ti_31_Nb_39_ was reported to display a high hydrogen permeability value of 1.93 × 10^−8^ mol m^−1^ s^−1^ Pa^−0.5^ at 400 °C, which is equivalent to the permeability of pure Pd in the same conditions [1,3].

The hydrogen permeation through a dense metal is a mass transfer phenomenon driven by the hydrogen pressure drop between the upstream and downstream walls of the metal [4]. The hydrogen permeation flux through a metal wall of thickness th is given by [4]:

(1) $$J=Pe\;\frac{\sqrt{P_{up}}-\sqrt{P_{down}}}{th}$$

where J is the hydrogen permeation flux (mol m^−2^ s^−1^), Pe is the permeability coefficient of hydrogen through the metal (mol m^−1^ s^−1^ Pa^−0.5^), $P_{up}$ and $P_{down}$ the hydrogen partial pressure (Pa) at the metal upstream and downstream surface, respectively, and  th (m) the thickness of the metal wall.

This expression of the hydrogen permeation flux is obtained by combining the Sieverts law and the Fick first law representing the hydrogen solubility in and the hydrogen diffusion through the metal lattice, respectively. In particular, according to the Sieverts law, the concentration of the hydrogen absorbed in the metal lattice is proportional to the square root of the hydrogen partial pressure in the gas phase in equilibrium with the metal [12]:

(2) $$c_{H}=k_{S}\sqrt{P}$$

where $c_{H}$ is the hydrogen concentration in the metal (mol m^−3^), $k_{s}$ the Sieverts constant (mol m^−3^ Pa^−0.5^) and P the hydrogen partial pressure in the gas phase (Pa). 

In parallel, one can write that the permeation flux (*J*) of hydrogen diffusing through the metal lattice is given by [4]:

(3) $$J=D\;\frac{c_{up}-c_{down}}{th}$$

where D is the diffusion coefficient (m^2^ s^−1^), *c_up_* and *c_down_* are the hydrogen concentration in the metal (mol m^−3^) at the upstream and downstream surface, respectively.

Combining Equations (2) and (3), the hydrogen permeation flux (*J*) can also be expressed as indicated in Equation (4).

(4) $$J=D\cdot k_{s}\frac{\sqrt{P_{up}}-\sqrt{P_{down}}}{th}$$

By combing Equations (1) and (4), it results that the hydrogen permeability coefficient, Pe, (mol m^−1^ s^−1^ Pa^−0.5^) can be estimated by the product of the diffusion, D, and solubility coefficients, $k_{s}$:

(5) $$Pe=k_{s}\;\cdot D$$

In view of Equation (5), if two of the previous physical quantities are known, the third one can be directly evaluated. Usually, from permeability and solubility measurements, the diffusion coefficient is derived, as reported in Refs. [13,14] for the Ni_30_Ti_30_Nb_40_ alloy. Permeability measurements, however, require specialized equipment, as well as a very demanding vacuum sealing between the material under examination and the test apparatus.

In this paper, we will explore the use of hydrogen absorption measurements to measure $k_{S}$ from the thermodynamics of the process and D from the kinetic response of the sample. From $k_{S}$ and D, the permeability of a Ni_33_Ti_39_Nb_28_ specimen will be derived. In the Sieverts apparatus used for this kind of measurement, there is no need for vacuum sealing on the sample; the only prerequisite for the derivation of the diffusion coefficient is a well-defined sample geometry, for which the Fick diffusion equations have already been solved in the literature. This method could be used at least as a pre-screening technique to evaluate the possibility of the application of a new alloy as a membrane for hydrogen purification.

In the present work, we will investigate an alloy with a slightly different composition from that reported by Hashi et al. [11] in order to enlarge the knowledge of this ternary system. Moreover, the temperature range between 400 and 500 °C, never explored before for Ni_33_Ti_39_Nb_28_ and similar alloys, will be investigated, as the maximum T of the previous measurements in Ni_30_Ti_31_Nb_39_ is 400 °C.

## 2. Results and Discussion

### 2.1. Alloy Microstructure

**Figure 1:**
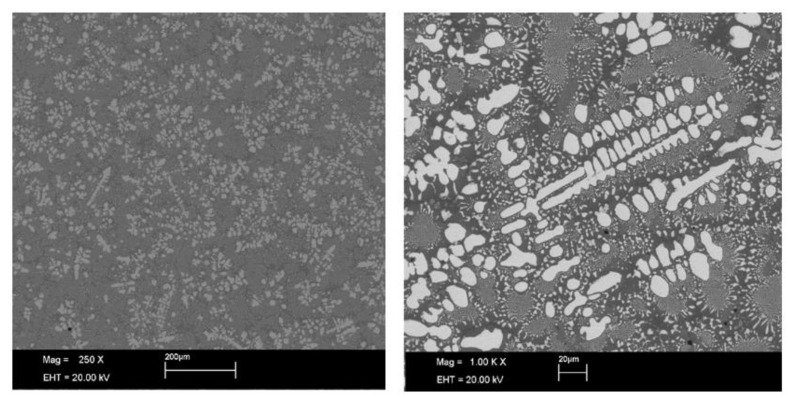
SEM micrographs of the as-cast Ni_33_Ti_39_Nb_28_ sample with different magnifications.

The microstructure of the presently investigated as-cast Ni_33_Ti_39_Nb_28_ is shown in Figure 1. In agreement with the previous studies of similar alloys with a slightly different elemental composition, the material is composed at the microscopic level of two interpenetrating phases: the white regions are the primary (Nb, Ti) solid solution phase, while the dark ones are the eutectic {(Nb, Ti) + TiNi} phases [13,15]. The micrographs also reveal two shades of dark color which, according to EDS spectroscopy, are related to the different content of the elements in the solid solution. In particular, the grey regions are characterized by a higher Ti content compared to the darker regions.

### 2.2. Hydrogen/Deuterium Absorption Isotherms

**Figure 2:**
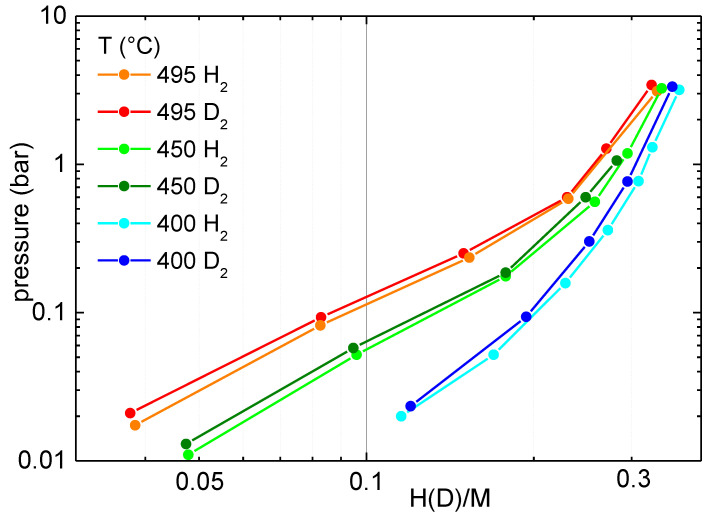
Pressure-composition isotherms in double logarithmic scale for hydrogen or deuterium absorption in Ni_33_Ti_39_Nb_28_ at selected temperatures.

Figure 2 reports the pressure-composition isotherms measured for the absorption of hydrogen and deuterium between 400 and 495 °C. In this figure, the pressure values are referred to the molecular hydrogen gas in equilibrium with the solid, while the values of the concentration are expressed as H(D)/M, i.e., the number of hydrogen/deuterium) atoms per metal atom composing the dehydrogenated alloy; these numbers are calculated by the ratio between the mass and the mass of the formula unit. One can observe in Figure 2 that at a fixed pressure, the concentration of hydrogen/deuterium decreases as temperature increases. The hydrogen isotherms are well comparable to those previously reported in Refs. [13,14] for temperatures between 300 and 400 °C. In general, during the hydrogenation (deuteration) of any material, there is an initial formation of a solid solution up to a certain hydrogen concentration, c_0_. For c < c_0_, the Sieverts law holds (p is proportional to c^n^), and there is no plateau. For c > c_0_, the hydride starts to form, and one can observe a plateau (even though, in many cases, it is not perfectly flat or horizontal). In Figure 2, one can observe two regions, one below the concentration value H(D)/M ≈ 0.2 and one above the value H(D)/M ≈ 0.25, where in both cases, the logarithm of the equilibrium pressure displays a linear dependence from the logarithm of the hydrogen/deuterium concentration, that is a region where the Sieverts law, p~c^n^, holds. For low values of the concentration, i.e., H(D)/M < 0.2, this corresponds to a region where a solid solution of hydrogen/deuterium in the crystalline matrix is present, while for higher values of H(D)/M the coexistence of a solid solution and the hydride (deuteride) starts to occur, even though the region of the plateau was not reached. For H(D)/M < 0.2, n=2.0 ± 0.1, so that the Sieverts law holds in its ideal form and no deviation from the ideal exponent (*n* = 2) is observed. In the presently investigated material, there is more than one phase: the bcc NbTi alloy and the eutectic phases NiTi and NbTi [11]. With the maximum pressure here used (p ≈ 4 bar), it is not possible to observe any pressure plateau at any of the explored temperatures because they are located at a pressure higher than the maximum pressure investigated here.

One can note in Figure 2 that the equilibrium pressure for hydrogen is slightly lower than for deuterium, even though this effect is extremely small. The occurrence of different equilibrium pressures for hydrogen and deuterium is due to the isotope effect, which in general can be either normal (p(H_2_) > p(D_2_)) or inverse (p(D_2_) > p(H_2_)). The typical examples of metals with normal and inverse isotope effects are V [16] and Pd [17], respectively, as well as most of their alloys [18,19]. The occurrence of either type of isotope effect was proven to be strictly linked to the presence of tetrahedral or octahedral interstitial sites for hydrogen or deuterium and to the different energies of these interstitials [20]. In the present case, the alloy is composed of Ni, Nb and Ti. Ti was reported to have a negligible isotope effect between 120 and 220 °C [21]; Nb displays a normal isotope effect at 60 °C [22], while Ni has an inverse isotope effect at 25 °C [23]. The presently investigated alloy shows a small inverse isotope effect, with p(D_2_) > p(H_2_). It must be noted that the temperature range of the present measurements is quite different from those reported in Refs. [21,22,23] and, recently, it was reported that temperature could play a fundamental role and transform materials from normal to inverse regarding their isotope effect [24]. Therefore, it is important to experimentally verify in a wide temperature range the possible presence of an isotope effect and its magnitude.

**Figure 3:**
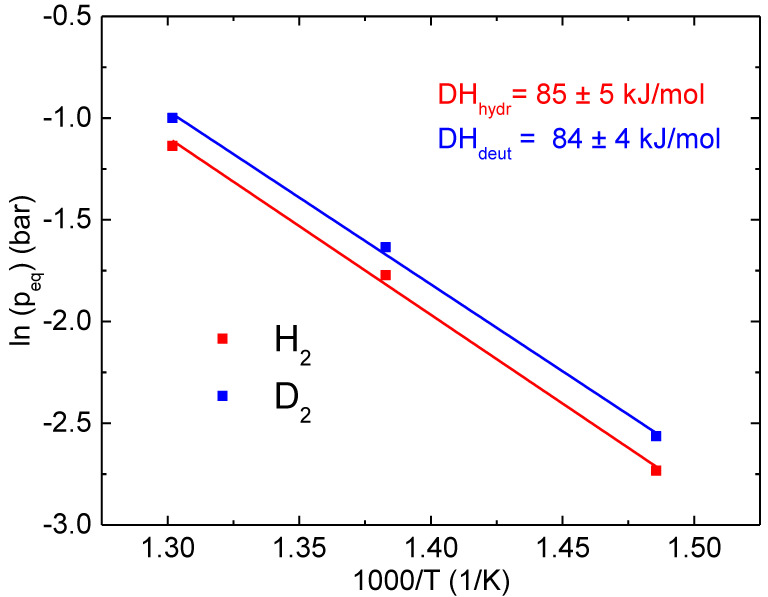
Van’t Hoff plot for Ni_33_Ti_39_Nb_28_ at the fixed H(D)/M of 0.18 and best fit lines to calculate the hydrogenation/deuteration enthalpy.

From the pressure-composition isotherms, it is possible to calculate the hydrogenation/deuteration enthalpy, ΔH(H_2_) and ΔH(D_2_), respectively, by means of a van’t Hoff plot at fixed H(D)/M. Figure 3 reports the van’t Hoff plot of the Ni_33_Ti_39_Nb_28_ alloy at the fixed composition H(D)/M = 0.18 and the best fit lines. The value H/M = 0.18 was chosen because it is in the region where the Sieverts law holds, and also, the data obtained at T = 400 °C was available (for lower concentrations, the equilibrium pressure at 400 °C was too low to be correctly measured with the presently used pressure transducers). The derived absolute values of the hydrogenation and deuteration enthalpy are ΔH(H_2_) = 85 ± 5 kJ/mol and ΔH(D_2_) = 84 ± 4 kJ/mol. Concerning the fits, an R^2^ of 0.995 and 0.997 was obtained for hydrogenation and deuteration, respectively. To the best of our knowledge, there was no previous report of the hydrogenation or deuteration enthalpy, even though the hydrogenation pressure-composition isotherms were already reported in Refs. [13,14].

The hydrogenation/deuteration enthalpy values here obtained for the Ni_33_Ti_39_Nb_28_ alloy are quite large, as compared to those reported for other materials proposed for hydrogen purification membranes. Pd_77_Ag_23_, indeed, was reported to have ΔH(H_2_) in the range 43–49 kJ/mol, depending on the temperature, and ΔH(D_2_) in the interval 32–43 kJ/mol [18,25]. The deuteration enthalpy of V_85_Ni_15_ is 47 kJ/mol [19], while for a series of amorphous ribbons with composition (Ni_0_._6_Nb_0_._4−y_Ta_y_)_100_ ΔH(H_2_) was ≈ 35–40 kJ/mol [26]. ΔH values comparable to those obtained for the Ni_33_Ti_39_Nb_28_ alloy were reported for Mg (ΔH(H_2_) ≈ 74.5 kJ/mol) [27] and an amorphous ribbon of Ni_32_Nb_28_Zr_30_Fe_10_ (ΔH(H_2_) ≈ 85 kJ/mol) [28].

### 2.3. Hydrogen/Deuterium Absorption Kinetics and Diffusion Coefficient

**Figure 4:**
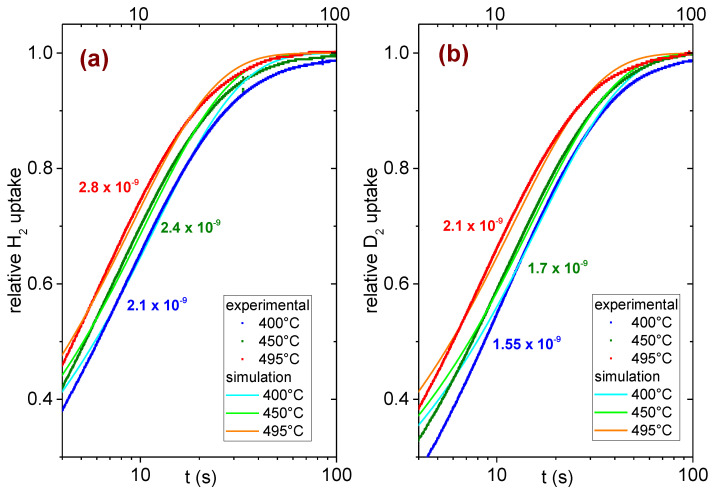
Time dependence of the relative absorption of hydrogen (panel (**a**)) or deuterium (panel (**b**)) at selected temperatures and best fit lines. The colored numbers are the derived coefficient of diffusion in m^2^/s.

Figure 4 displays the time evolution of the uptake of hydrogen/deuterium for the Ni_33_Ti_39_Nb_28_ alloy at 400, 450 and 495 °C. In all cases, an initial pressure of 1 bar was applied to the sample. As the kinetics is quite fast (90% of the hydrogenation occurs in less than 30 s, see Figure 4), the signal coming from the pressure transducer was acquired by means of a Keithley DAQ6510 multimeter system that recorded the output voltage every 1/50 s. It is well evident in Figure 4 that the kinetics becomes faster as the temperature increases and that the alloy absorbs hydrogen faster than deuterium.

The sample that we used for the measurements had a well-defined parallelepiped shape, with dimensions of 0.51 × 12.0 × 28.0 mm^3^ (an uncertainty of 1 unit on the last reported digit should be considered). Due to the occurrence of this specific shape, it was possible to derive the diffusion coefficient from the kinetics of the hydrogen/deuterium absorption under reasonable approximations. Indeed, the equations for the diffusion of atoms in solids were solved many years ago, and various geometries and boundary conditions were considered. In the present case, since one of the dimensions of the sample is much smaller than the others (by a factor of at least 24), the specimen can be assimilated to a 1D slab of thickness 0.51 mm, and the diffusion process can be considered to occur along this thickness (therefore perpendicularly to the large face of the samples of dimensions 12.0 mm × 28.0 mm). Under these conditions, for times sufficiently high, the absorbed quantity of hydrogen/deuterium ($M_{t}$) (mol) with respect to the total quantity at the infinite time ($M_{\infty }$) (mol) is [29,30]:

(6) MtM∞=1−∑n=0∞8(2n+1)2π2exp{−D(n+12)2π2tl2}

where t is time, D the diffusion coefficient and 2l the thickness of the sample. The convergence of the infinite series is quite fast for sufficiently long times, and indeed already, the sum of the first two terms of the series is sufficient to describe the shape of the absorption curves of Figure 4 for t > 5 s. We verified that no change occurs in the curves for t > 5 s, even adding other terms of the sum of Equation (5).

In Figure 4, the curves obtained from Equation (5) that best approximate the experimental curves are reported, together with the obtained values of the diffusion coefficient. They describe the experimental data for t > 5 s, corresponding to the range $0.4<\frac{M_{t}}{M_{\infty }}<0.95$. The derived diffusion coefficients are in the range of a few 10^−9^ m^2^/s and are in line with those reported for T ≤ 400 °C [13,14]. As expected, the diffusion coefficient increases with the temperature; moreover, D is higher for hydrogen than for deuterium.

The diffusion coefficients of the presently investigated alloy are slightly lower than those of Pd-based membranes at the same temperatures that are the standard membrane for hydrogen purification. Indeed, it was reported and analyzed that Pd-based membranes display a diffusion coefficient ranging between 5 and 10 × 10^−9^ m^2^/s in the temperature range between 400 and 500 °C [31]. The D values of the Ni_33_Ti_39_Nb_28_ alloy are also similar to those of V [32] and Pd-V alloys [33].

**Figure 5:**
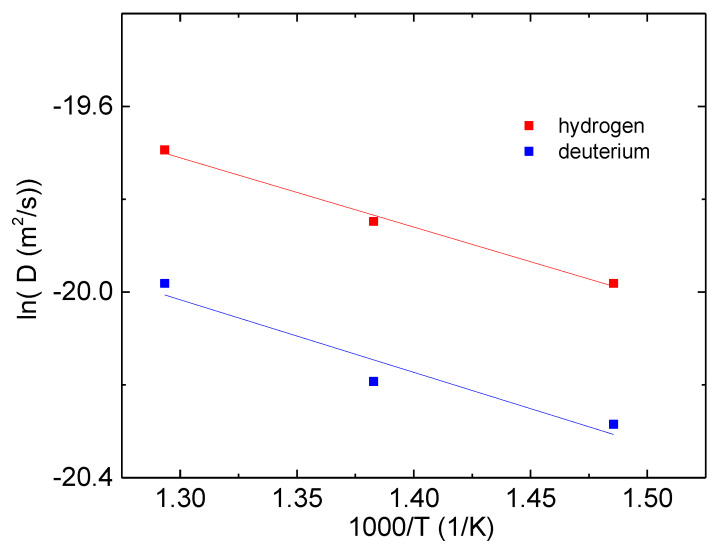
Dependence of the logarithm of the calculated diffusion coefficient of Ni_33_Ti_39_Nb_28_ from the inverse of the absolute temperature and best fit lines.

The temperature dependence of the diffusion coefficient usually follows an Arrhenius exponential law with activation energy, E_a,d_(H_2_) or E_a,d_(D_2_) (kJ/mol), describing the energy barrier that hydrogen or deuterium atoms should overcome in order to diffuse in the solid. Figure 5 reports the logarithm of D for hydrogen and deuterium as a function of the inverse of the absolute temperature. From the fit to a linear regression, one obtains a diffusion activation energy E_a,d_ = 12 ± 1 kJ/mol for both hydrogen isotopes (R^2^ = 0.986 for hydrogenation and 0.88 for deuteration). This value is smaller than that found for similar alloys [13,14], which, on the other hand, ranged between 25 and 30 kJ/mol for the explored temperature range between 300 and 400 °C. It must be said that both the temperature range considered in Refs. [13,14] and the composition of the studied alloys are slightly different from those investigated here, and this can at least partially explain the difference in E_a,d_.

It is important to note that the investigation of the diffusion coefficient in the present case was conducted thanks to measurements of the kinetics of hydrogen/deuterium absorption, that is, using a much simpler procedure than the evaluations from the permeation experiments, which requires a demanding vacuum tight welding of the used membrane. In the present experiments, the only prerequisite for obtaining a reliable diffusion coefficient is the use of a sample shaped with a well-defined geometry. For the investigated sample, the geometry was that of a parallelepiped with one of the dimensions much smaller than the other; however, well established equations for the diffusion process are also available for other geometries, such as parallelepipeds with generic dimensions, cylinders and spheres [29,30].

### 2.4. Assessment of Hydrogen Isotope Permeation

From Equations (1)–(5) and the measurements of the solubility and diffusion coefficient of the Ni_33_Ti_39_Nb_28_ alloy, the permeability of the material was derived. In view of the temperature dependence of the solubility and diffusivity, the permeability coefficient Pe (mol m^−1^ s^−1^ Pa^−0.5^) is expected to display an Arrhenius dependence on T:

$$Pe=Pe_{o\;}e^{-\frac{Ea,p}{T}}$$

where $Pe_{0}$ is the pre-exponential factor (mol m^−1^ s^−1^ Pa^−0.5^) and $E_{a,p}$ the activation energy (K^−1^).

**Figure 6:**
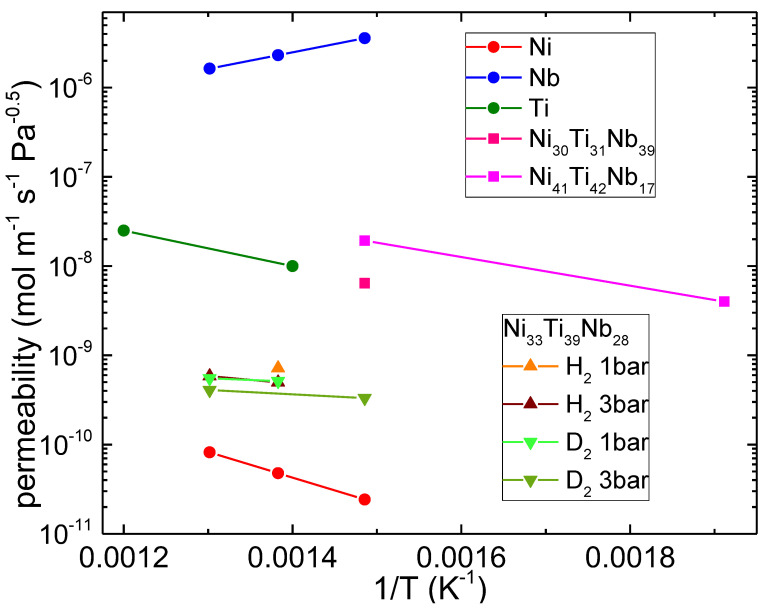
Calculated hydrogen and deuterium permeability for the presently investigated Ni_33_Ti_39_Nb_28_ alloy and comparison with the literature (Ni [10], Nb [10], Ti [34], Ni_30_Ti_31_Nb_39_ [11] and Ni_41_Ti_42_Nb_17_ [11]).

The calculated permeability is shown in Figure 6 and reported in Table 1. From the diffusion and solubility data, the permeability of the alloy has been evaluated at around 1 bar and around 3 bar. A comparison with literature data is also reported.

In general, the permeability of the studied alloy decreases with the pressure, while it increases with the temperature. This temperature dependence resembles the behavior of Ni and Ti (E_a,p_ > 0) and is opposite to that of the Nb, whose permeability reduces with increasing temperature (E_a,p_ < 0). The permeability of the material studied in this work stays between the values reported in the literature for Ni and Ti. The alloy Ni_30_Ti_29_Nb_41_ described by Hashi has a higher Nb content (41 at%) and shows a higher hydrogen permeability [11], although the temperature dependence is still that of Ni and Ti (E_a,p_ > 0). The other alloy reported by Hashi, Ni_41_Ti_42_Nb_17_, has a reduced amount of Nb (17 at%) and exhibits permeability values in line with those of Ti [11], which, in fact, is its major component.

The values of the permeability of the presently investigated alloy are one order of magnitude lower than those of the best performing Ni–Ti–Nb alloy at the same temperature; however, it must be noted that the permeability of Ni_33_Ti_39_Nb_28_ is only one order of magnitude lower than that of Pd at the same T (Pe = 1.8 × 10^−8^ molm^−1^ s^−1^ Pa^−0.5^ [1]), which is the industry standard for hydrogen purification by means of reactors.

## 3. Materials and Methods

The Ni_33_Ti_39_Nb_28_ ingot was prepared at the RINA Consulting—Centro Sperimentale Materiali Institute by using the vacuum induction melting (VIM) technique, which is one of the most versatile melting processes to produce almost all special alloys based on Fe, Ni and Co. The VIM technique uses an alternating current passing through a coil to generate eddy currents in the materials to be synthesized so that they have melted, thanks to the Joule effect. Continuous stirring is applied to the bath to improve chemical and temperature homogenization of the melt and to favor degassing. The melting cycle in a VIM furnace is usually structured in several steps: filling, melting, refining, chemical analysis, composition correction and casting. The filler material includes the binding elements, except for the reactive constituents, which are subsequently introduced by means of a system placed on the top of the furnace. During melting and refining, reactions such as degassing and deoxidation take place. At the end of the refining period, reactive elements (like Al, Ti, Zr, B) are introduced, if necessary. After a certain time required for the optimal mixing of the bath, chemical analyses are conducted, and, in case of deficiencies, alloying elements are added to the composition. In the end, the melt is poured into an ingot mold.

**Figure 7:**
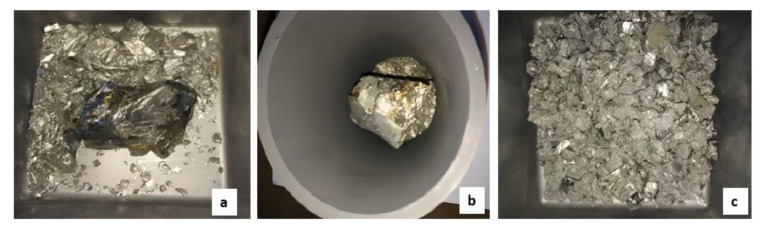
(**a**) initial NbNi sample, (**b**) NbNi alloy in the graphite crucible and (**c**) Ti “sponge”.

In the present case, raw materials with a purity not lower than 99.9% were used for the filler materials and melted in an isostatic graphite crucible to form the Nb–Ni–Ti ingot. The fabrication process was conducted in several steps. At first, Ni and Nb were loaded in the crucible, washed with argon several times, and evacuated at 1 Pa. In these vacuum conditions, the furnace temperature was raised to melt the charged materials. After melting, the material was continuously stirred for ~30 min. Afterward, the required amount of pure Ti was added to reach the desired composition. The addition of Ti occurs later because of its high reactivity. The final temperature of the process was 1370 °C. Pictures of the initial Ni and Nb elements are provided in Figure 7a,b, while 7c displays the Ti material added in a second time.

**Figure 8:**
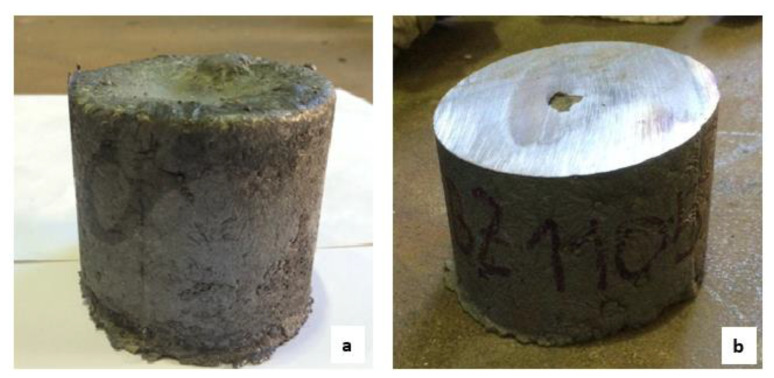
(**a**) Ni_33_Ti_39_Nb_28_ ingot as removed from the crucible, (**b**) Ni_33_Ti_39_Nb_28_ ingot after cutting.

After the VIM process, the Ni_33_Ti_39_Nb_28_ ingot, with a diameter of 80 mm, was removed from the crucible. Figure 8 shows the ingot before and after cutting; its final measured composition is reported in Table 2.

**Figure 9:**
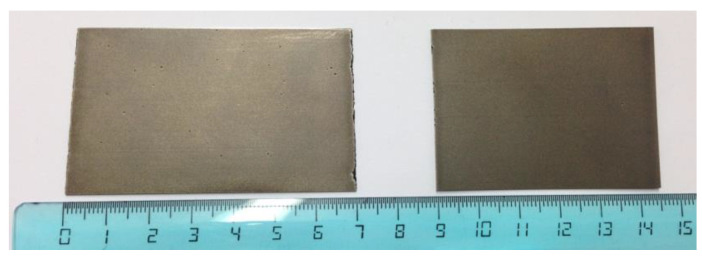
Picture of the two Ni_33_Ti_39_Nb_28_ sheets obtained after electroerosion.

From the ingot, a foil of 11 mm has been obtained and treated by hot rolling cycles at 1150 °C. Theoretically, after 11 rolling cycles, the foil should have reached a thickness of 3 mm. However, after a reduction of about 20% (corresponding to 3 rolling cycles), the foil disintegrated, with several cracks passing the entire thickness. Therefore, it was decided to extract via electroerosion two sheets having a thickness of 0.5 mm (see Figure 9), from which the sample used for the presently reported measurements was cut.

The measure of the absorption of hydrogen or deuterium was conducted by means of a homemade Sieverts apparatus described in Ref. [35]. A single piece of the Ni_33_Ti_39_Nb_28_ alloys with a parallelepiped shape with dimensions 0.51 × 12.0 × 28.0 mm was used for all the measurements. In order to activate the surface, it was first heated up to 490 °C and maintained in a vacuum at this temperature overnight; afterward, it was subject to three cycles of hydrogen charge/discharge. Hydrogen was removed from the solid sample by means of a turbopump that allowed it to reach a vacuum of the order of 10^−4^ mbar in the sample holder during an overnight treatment. The temperature range was selected in order to complement the measurements available in the literature on Ni–Ti–Nb alloys with similar composition, which extended from 300 to 400 °C [11,13,14].

By means of the Sieverts apparatus, it was possible to measure pressure-composition curves at 495, 450 and 400 °C in the pressure range below 4 bar. The same sample was used for all measurements, which were duplicated to check the reproducibility. The uncertainties on the single measurements were smaller than the points reported in the figures. The uncertainties on the enthalpy values were obtained from the fits. Finally, the kinetics of hydrogenation/deuteration was monitored at each of these temperatures using a Keithley DAQ6510 multimeter acquisition system that recorded the fast time evolution of the pressure transducer voltage. For each acquisition, an initial pressure of 1 bar of either hydrogen or deuterium was applied to the sample. The analysis of the experimental data and the simulations of the absorbed hydrogen were performed by means of the software Origin Pro [36].

## 4. Conclusions

In summary, a Ni_33_Ti_39_Nb_28_ sample was synthesized by the vacuum induction melting technique starting from pure metals. Samples with a regular shape have been obtained via electroerosion. The material was very brittle, and it was not possible to further reduce the thickness via hot rolling. The pressure-composition isotherms for the absorption of hydrogen and deuterium were measured in the temperature range between 400 and 495 °C and for pressure lower than 3 bar. For hydrogen concentrations H(D)/M < 0.2, the Sieverts law is valid with an exponent of 2.0 ± 0.1. At higher concentrations, a deviation is observed. The hydrogenation/deuteration enthalpy absolute values are ΔH(H_2_) = 85 ± 5 kJ/mol and ΔH(D_2_) = 84 ± 4 kJ/mol. The kinetics of absorption of both hydrogen isotopes was recorded, and thanks to the parallelepipedal shape of the sample, the diffusion coefficient at selected temperatures was derived. An Arrhenius-type dependence from the temperature was obtained, with E_a,d_ = 12 ± 1 kJ/mol for both hydrogen isotopes. Finally, from the knowledge of both solubility and diffusion coefficient, an estimate of the permeability of this previously unknown alloy is obtained. In the temperature range between 400 and 495 °C the permeability is of the order of a few units × 10^−9^ mol m^−1^ s^−1^ Pa^−0.5^, a value which is one order of magnitude lower than that of Ni_41_Ti_42_Nb_17_, until now the best performing Ni–Ti–Nb alloy for hydrogen purification. All the physical quantities reported in the present paper were obtained by means of sorption measurements performed in a Sieverts apparatus. This method can be useful for the preliminary screening of new alloys to derive their permeability without the need for a dedicated apparatus.

## Figures and Tables

**Table 1:** Hydrogen and deuterium permeability of Ni_33_Ti_39_Nb_28_ calculated at selected temperatures and pressures and comparison with the literature.

	Pe_0_ mol m^−1^ s^−1^ Pa^−0.5^	E_a,p_ K^−1^	T K	P kPa	Ref.
Ni_33_Ti_39_Nb_28_, D_2_	1.86 × 10^−9^	934	673–768	100	this work
Ni_33_Ti_39_Nb_28,_ H_2_	7.34 × 10^−9^	1945	673–768	300	this work
Ni_33_Ti_39_Nb_28_, D_2_	1.74 × 10^−9^	1113	673–768	300	this work
Ni_30_Ti_31_Nb_39_, H_2_	4.67 × 10^−6^	3741	523–673	200–970	[11]
Ni, H_2_	4.65 × 10^−7^	6640	650–920	10–100	[10]
Nb, H_2_	6.30 × 10^−9^	−4270	>673	>10	[10]
Ti, H_2_	6.10 × 10^−6^	4581	673–950	-	[34]

**Table 2:** Composition of the Ni-Nb-Ti sample (in wt% and at%).

	Ni	Nb	Ti
wt%	40.69	30.30	29.01
at%	33.09	28.07	38.84

## Data Availability

Data are contained within the article.

## Sample Availability

Samples of the compound are available from the authors.
